# Toward a physics-guided machine learning approach for predicting chaotic systems dynamics

**DOI:** 10.3389/fdata.2024.1506443

**Published:** 2025-01-17

**Authors:** Liu Feng, Yang Liu, Benyun Shi, Jiming Liu

**Affiliations:** ^1^Department of Computer Science, Hong Kong Baptist University, Hong Kong, China; ^2^College of Computer and Information Engineering, Nanjing Tech University, Nanjing, China

**Keywords:** physics-guided, data-driven, deep learning, chaotic systems, dynamics prediction

## Abstract

Predicting the dynamics of chaotic systems is crucial across various practical domains, including the control of infectious diseases and responses to extreme weather events. Such predictions provide quantitative insights into the future behaviors of these complex systems, thereby guiding the decision-making and planning within the respective fields. Recently, data-driven approaches, renowned for their capacity to learn from empirical data, have been widely used to predict chaotic system dynamics. However, these methods rely solely on historical observations while ignoring the underlying mechanisms that govern the systems' behaviors. Consequently, they may perform well in short-term predictions by effectively fitting the data, but their ability to make accurate long-term predictions is limited. A critical challenge in modeling chaotic systems lies in their sensitivity to initial conditions; even a slight variation can lead to significant divergence in actual and predicted trajectories over a finite number of time steps. In this paper, we propose a novel Physics-Guided Learning (PGL) method, aiming at extending the scope of accurate forecasting as much as possible. The proposed method aims to synergize observational data with the governing physical laws of chaotic systems to predict the systems' future dynamics. Specifically, our method consists of three key elements: a data-driven component (DDC) that captures dynamic patterns and mapping functions from historical data; a physics-guided component (PGC) that leverages the governing principles of the system to inform and constrain the learning process; and a nonlinear learning component (NLC) that effectively synthesizes the outputs of both the data-driven and physics-guided components. Empirical validation on six dynamical systems, each exhibiting unique chaotic behaviors, demonstrates that PGL achieves lower prediction errors than existing benchmark predictive models. The results highlight the efficacy of our design of data-physics integration in improving the precision of chaotic system dynamics forecasts.

## 1 Introduction

Chaotic systems are ubiquitous, from academic research in physics (Pecora and Carroll, 1990; Grassberger and Procaccia, 1983) and chemistry (Hess, 1990; Field et al., 1993) to real-world domains such as epidemiology (Aguiar et al., 2008; Mishra et al., 2020) and climatology (Palmer, 1993; Olsen et al., 2019). By predicting the dynamics of these systems, we can gain valuable insights into their future behaviors, which can not only help us understand the underlying mechanisms of these systems but, more importantly, effectively inform and guide the decision-making process in real-world problems within the respective fields. For example, forecasting the dynamical behaviors in the spread of epidemics can help us uncover the disease transmission patterns and, accordingly, deploy effective intervention strategies to control infectious diseases (Mangiarotti et al., 2016). Predicting the dynamics of variables in the climate system, such as temperature and precipitation, can help us be well prepared for extreme weather events (Toreti et al., 2013).

In recent years, with the availability of large amounts of data and the advancement of computing power, many studies have utilized data-driven approaches to analyze and predict the dynamics of chaotic systems. These methods generally utilize the given data to learn the mapping function between historical observations and the future value of the target variable, and then use the learned mapping function to conduct the prediction. Typical data-driven methods that have been widely used in chaotic system dynamics prediction include long short-term memory networks(LSTM) (Hochreiter, 1997; Chattopadhyay et al., 2020), reservoir computing (Jaeger, 2001; Pathak et al., 2018), etc. The above methods have been proven to be effective for the short-term prediction of chaotic systems, demonstrating an ability to capture the instantaneous dynamics (Chantry et al., 2021). However, their ability to make long-term predictions is limited, especially for those rapidly evolving chaotic dynamical systems, where even a slight initial variation can result in significant differences as the evolution over time (Lorenz, 1963). The reason could be that such data-driven methods rely solely on historical observations during the learning process but ignore the underlying mechanisms of chaotic systems, which are, in fact, of great importance in characterizing the systems' dynamical behaviors.

To overcome the limitations of pure data-driven models in predicting chaotic system dynamics and to enhance prediction performance, several existing studies have combined data with physical mechanisms. For example, PIESN (Doan et al., 2020) and its variant (Na et al., 2023) encode the systems' governing equations into the models' loss functions, penalizing predictions that deviate from physical laws. Furthermore, other methods utilize physical knowledge to help reconstruct and predict the dynamics of chaotic systems with unmeasured variables (Racca and Magri, 2021; Özalp et al., 2023). These methods, however, typically require complete and precise knowledge of the governing differential equations of the systems, including the equation parameters, to effectively guide the predictive models, which limits their applicability. Meanwhile, the reconciliation between data-driven approaches and prior physical knowledge remains an open yet essential problem in the prediction of chaotic systems' dynamics.

To effectively extend the capability for chaotic dynamics prediction, in this paper, we introduce a novel method called Physics-Guided Learning (PGL). Inspired by a recently developed physics-informed neural network (PINN), which was originally designed for solving forward and reverse problems in nonlinear partial differential equations (Raissi et al., 2019), our PGL method seeks to synergize observational data with the governing physical laws of chaotic systems. In our study, we operate under the assumption that the knowledge of the dynamical system we aim to predict is partially available. Specifically, we assume familiarity with the structure of the ordinal differential equations, while the parameters of these equations remain unknown and will be inferred throughout the learning process. This modest assumption has been widely adopted in recent research in physics-informed machine learning and aligns with many real-world scenarios where precise governing equations are not accessible (Misyris et al., 2020; Nath et al., 2023; Ning et al., 2023). For example, in climate modeling, researchers often rely on the well-established Navier-Stokes equations, despite the challenges in determining their exact parameters and solutions (Yang et al., 2023; Gao et al., 2024). The architecture of PGL is composed of three integral components: a data-driven component that learns the dynamical patterns and mapping functions from historical observations, a physics-guided component that exploits and represents systems' governing mechanisms, and a nonlinear learning component that integrates the output from the data-driven component and that from the physics-guided component in a proper way. The objective functions of these three components will be jointly optimized to achieve the desired goal of chaotic dynamics prediction.

Several related works have explored the use of neural networks to generate chaotic dynamics. Notably, Hopfield Neural Networks (Hopfield, 1984) with memristors (Chua, 1971) have attracted much attention due to their flexible network architecture and bio-inspired characteristics. These models have been employed to produce a variety of chaotic dynamics, including multi-scroll, coexisting, and hyperchaotic attractors (Li et al., 2022; Kong et al., 2024; Deng et al., 2024). In contrast to approaches that generate dynamics with chaotic characteristics for applications such as image encryption (Liu et al., 2019) and privacy protection (Hu et al., 2024), and that do not necessitate reference to a specific dynamical system, our study seeks to predict the dynamical behaviors of a particular chaotic system. We employ data-driven methods, specifically neural networks, leveraging historical observations and partial knowledge of the chaotic system being modeled. By integrating data with physical principles, we aim to extend the scope and accuracy of chaotic dynamics prediction.

The remainder of this paper is organized as follows. Section 2 outlines the proposed methodology, with a detailed explanation of its core principles, architecture design, and learning processes. In Section 3, we present the settings and results of our experiments on six typical chaotic systems, which are designed to validate the effectiveness of the proposed method in the task of chaotic dynamics prediction. Finally, we conclude our work in Section 4.

## 2 Methodology

In this section, we will outline the formalism and computational mechanism of the proposed PGL method. We begin by defining the learning problem and providing an overview of the method. Subsequently, we present the mathematical definition and formulation of the proposed method for chaotic system dynamics prediction, which integrates data and physical understanding. To enhance the clarity, we detail the method's structure, workflow, and objective function.

### 2.1 Problem statement

First, we state the definition of chaotic system dynamics prediction. For a chaotic system with *N* state variables, we represent the system's state observations at time *t* as ${\text{X}}_{t}=\left[x_{t}^{1},x_{t}^{2},\ldots ,x_{t}^{N}\right]$. **X**_*t*−*L*+1:*t*_ = [**X**_*t*−*L*+1_, **X**_*t*−*L*+2_, …, **X**_*t*_] denotes the historical data containing *L* time steps. Meanwhile, the time point sequence **T**_*t*−*L*+1:*t*_ = [*t*−*L*+1, *t*−*L*+2, ⋯*t*] corresponding to the system's state value sequence **X**_*t*−*L*+1:*t*_ is also recorded. The target of chaotic system dynamics prediction is to learn the underlying state transition function and the potential dynamics of the system based on the historical data and governing physical laws, and then forecast the subsequent state of the chaotic system, denoted as ${\tilde{\text{X}}}_{t+1}$. To achieve this goal, we devise a PGL method that makes use of both the observational data and the underlying dynamical mechanism of the chaotic system. Specifically, the proposed method comprises three core components: a data-driven component (DDC), a physics-guided component (PGC), and a nonlinear learning component (NLC). In the subsequent section, we will furnish a more detailed exposition of our design.

### 2.2 Physics-guided learning

**Figure 1:**
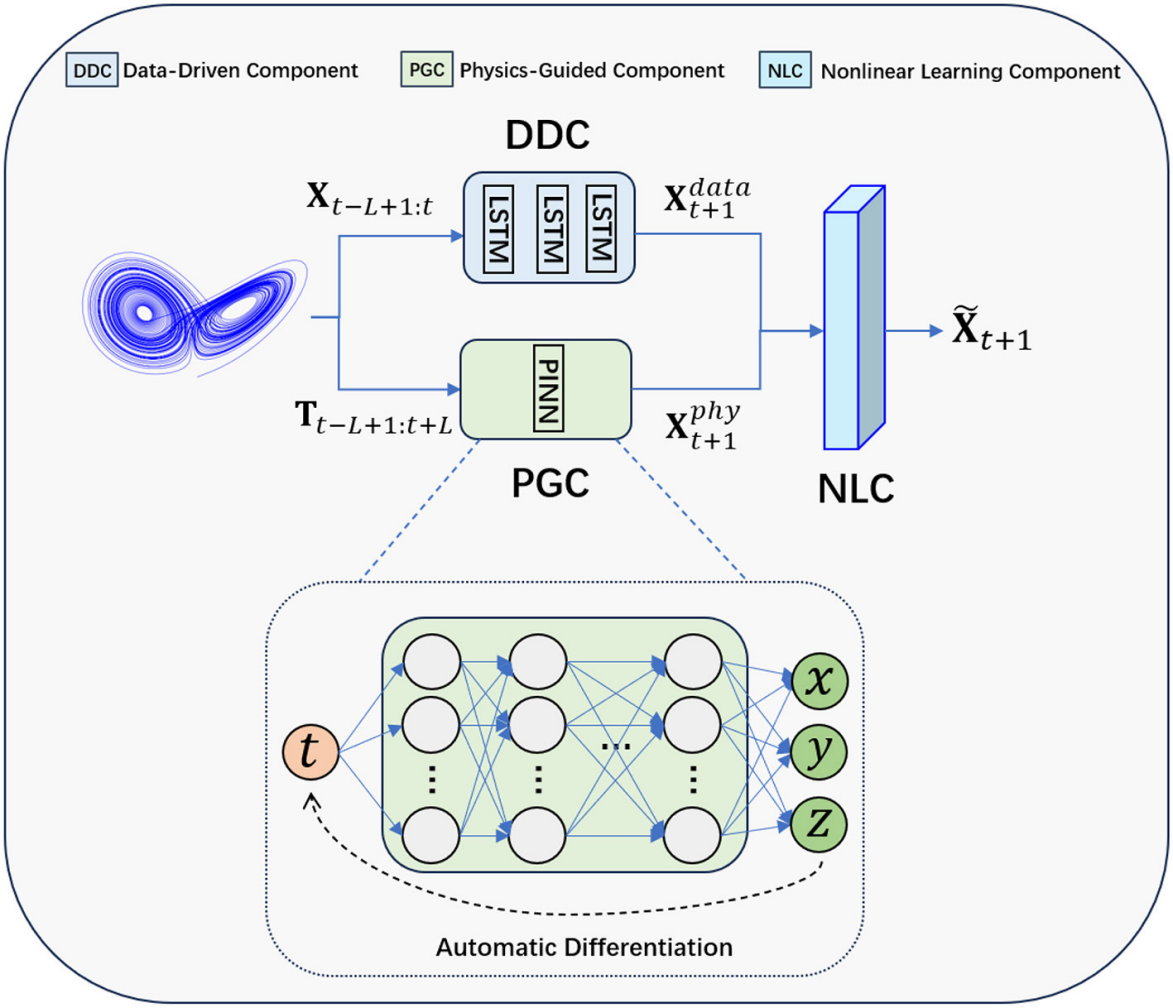
Illustration of the architecture of the proposed method PGL, which is composed of three core components: a data-driven component (DDC), a physics-guided component (PGC), and a nonlinear learning component(NLC).

Figure 1 illustrates the architecture of the proposed method PGL, consisting of DDC, PGC, and NLC. For the DDC, we use a three-layer LSTM with 20 hidden units each, followed by a dense layer. For the PGC, we refer to the PINN configuration (Raissi et al., 2019), using a 10-layer neural network with 32 neurons in each layer. For the third component NLC, note that it is intentionally designed to affirm the feasibility of the proposed idea of integrating data-driven and physics-guided components. Due to the real-world data often exhibits different complex nonlinear patterns, our model, which can be seen as a physics-guided learning framework, is designed with flexibility, allowing for the incorporation of different sophisticated neural network architectures to accommodate and adapt to these higher levels of complexity. In this paper, we utilize two typical architectures–the multi-layer perceptron (MLP)^1^ and the attention mechanism–as examples to demonstrate our design of the NLC. Specifically, the MLP-based NLC has two layers: one input layer and one output layer. In the attention-based NLC, we utilize the cross-attention mechanism to capture the nonlinearity in the DDC and PGC's outputs (Vaswani, 2017; Shi et al., 2024). Note that other deep learning modules or architectures can also be flexibly integrated into our framework as the NLC. Next, we will elaborate in detail on how these three components work together to predict the dynamical behaviors of chaotic systems.

#### 2.2.1 Data-driven component

Firstly, we obtain the prediction of the data-driven branch for the next time step, denoted by ${\text{X}}_{t+1}^{data}=DDC\left({\text{X}}_{t-L+1:t}\right)$. We expect the long short-term memory (LSTM) structure in the DDC to capture both short-term and long-term temporal dependencies in the historical state sequence through its unique gating mechanism and make predictions for the next time step.

#### 2.2.2 Physics-guided component

Afterward, we extend the **T**_*t*−*L*+1:*t*_, turning it into **T**_*t*−*L*+1:*t*+*L*_, which is further fed into the PGC. The PGC generates the system state predictions that are of equal length to the extended time sequence **T**_*t*−*L*+1:*t*+*L*_. This process is shown in the following equation:


(1) $$\begin{array}{l} {\text{X}}_{t-L+1:t+L}^{phy}=PGC\left(t-L+1,t-L+2,...,t+L\right), \end{array}$$

where ${\text{X}}_{i}^{phy}=\left[x_{i}^{phy},y_{i}^{phy},z_{i}^{phy}\right]$. We expect that, with the guidance of physical knowledge, the PGC can learn the dynamics of the system and assist the entire model in making predictions. Note that the design of PGC is general and can be used in various chaotic systems. Here, for a better explanation, we use the typical Lorenz system (Lorenz, 1963) as an example to show how the PGC works. The only information that we have is the form of the system's equations shown in the following Equation 2, and we do not know the crucial initial values and system parameters.


(2) $$\begin{array}{l} \frac{dx}{dt}=a\left(y-x\right),\\ \frac{dy}{dt}=cx-y-xz,\\ \frac{dz}{dt}=xy-bz. \end{array}$$

Following the work of physics-informed neural networks in Raissi et al. (2019), we utilize the automatic differentiation tools within the deep learning framework PyTorch (Paszke et al., 2017) to compute the derivative of the PGC's output ${\text{X}}_{t-L+1:t+L}^{phy}$ with respect to its input **T**_*t*−*L*+1:*t*+*L*_, yielding the following:


(3) $$\begin{array}{l} \frac{\partial {\text{X}}_{t-L+1:t+L}^{phy}}{\partial t}=\left[\frac{\partial {\text{X}}_{t-L+1}^{phy}}{\partial t},\frac{\partial {\text{X}}_{t-L+2}^{phy}}{\partial t},\cdots \;,\frac{\partial {\text{X}}_{t+L}^{phy}}{\partial t}\right], \end{array}$$

where $\frac{\partial {\text{X}}_{i}^{phy}}{\partial t}=\left[\frac{\partial x_{i}^{phy}}{\partial t},\frac{\partial y_{i}^{phy}}{\partial t},\frac{\partial z_{i}^{phy}}{\partial t}\right]$. We expect that the approximate derivatives conform to the definition of the Lorenz system, and therefore, we have calculated the residuals with respect to the physics-guided component, as shown below.


(4) $$\begin{array}{l} loss_{phy}=\lambda_{1}loss_{x}+\lambda_{2}loss_{y}+\lambda_{3}loss_{z},\\ loss_{x}=\overset{t+L}{\underset{i=t-L+1}{\sum }}|\frac{\partial x_{i}^{phy}}{\partial t}-ã\left(y_{i}^{phy}-x_{i}^{phy}\right){|}^{2},\\ loss_{y}=\overset{t+L}{\underset{i=t-L+1}{\sum }}|\frac{\partial y_{i}^{phy}}{\partial t}-\left(\tilde{c}x_{i}^{phy}-y_{i}^{phy}-x_{i}^{phy}z_{i}^{phy}\right){|}^{2},\\ loss_{z}=\overset{t+L}{\underset{i=t-L+1}{\sum }}|\frac{\partial z_{i}^{phy}}{\partial t}-\left(x_{i}^{phy}y_{i}^{phy}-\tilde{b}z_{i}^{phy}\right){|}^{2}, \end{array}$$

where λ_1_, λ_2_, and λ_3_ are hyper parameters which can be selected by a grid search strategy from a predefined rough range in practice. ã, $\tilde{b}$, and $\tilde{c}$ are trainable parameters of the model. Note that the true parameters of the chaotic systems remain unidentified for the PGC and for the proposed PGL model, a scenario that is typical in real-world applications. It is our expectation that the proposed model is capable of learning and characterizing the systems' dynamics even in the presence of such uncertainties. Additionally, since we have the ground truth **X**_*t*−*L*+1:*t*_, we conduct supervised learning by minimizing the following *loss*_*data*_:


(5) $$\begin{array}{l} loss_{data}=\frac{1}{L}\overset{t}{\underset{i=t-L+1}{\sum }}|{\text{X}}_{i}^{phy}-{\text{X}}_{i}{|}^{2}. \end{array}$$

By incorporating penalty terms based on physics and data, we hope that the PGC can rely on known physical knowledge and work in collaboration with the DDC to predict chaotic systems.

#### 2.2.3 Nonlinear learning component

Next, a nonlinear learning component will balance the predicted ${\text{X}}_{t+1}^{data}$ and ${\text{X}}_{t+1}^{phy}$ from DDC and PGC to provide the final prediction ${\tilde{\text{X}}}_{t+1}$ for the system at the time step *t*+1. In the following, we will introduce the MLP-based NLC and the Attention-based NLC, separately.

##### 2.2.3.1 MLP-based NLC

In the MLP-based NLC, we utilize a classical structure of MLP to conduct the nonlinear learning task, which can be described as the following equation:


(6) $$\begin{array}{l} {\tilde{\text{X}}}_{t+1}=NLC\left(concatenate\left({\text{X}}_{t+1}^{data},{\text{X}}_{t+1}^{phy}\right)\right), \end{array}$$

where ${\tilde{\text{X}}}_{t+1}$ represents the predicted value for the next time step. To constraints the learning process, we also calculate the loss which is formulated as follows:


(7) $$\begin{array}{l} loss_{NLC}=|{\tilde{\text{X}}}_{t+1}-{\text{X}}_{t+1}{|}^{2}, \end{array}$$

where **X**_*t*+1_ denotes the ground truth value of the system's state variable at time step *t*+1, which serves as the label in our supervised learning. It is important to note that the data for **X**_*t*+1_ in Equation 7 is exclusively accessible during the training phase. This information is not available during the testing phase, where the model must predict **X**_*t*+1_ without the aid of ground truth values.

##### 2.2.3.2 Attention-based NLC

In the Attention-based NLC, we use a specifically designed attention mechanism, i.e. the cross-attention, to learn the nonlinearity and make the final predictions. First, the attention mechanism generates the query *Q*_*data*_, the key *K*_*data*_, and the value *V*_*data*_ by applying linear transformations to ${\text{X}}_{t+1}^{data}$, i.e., $Q_{data}={\text{X}}_{t+1}^{data}\cdot W_{q}^{data}$, $K_{data}={\text{X}}_{t+1}^{data}\cdot W_{k}^{data}$, and $V_{data}={\text{X}}_{t+1}^{data}\cdot W_{v}^{data}$, where $W_{q}^{data}$, $W_{k}^{data}$, and $W_{v}^{data}$ are the trainable matrices. Similarly, we can obtain the query *Q*_*phy*_, the key *K*_*pyh*_, and the value *V*_*phy*_ by performing the same calculation for the output of PGC ${\text{X}}_{t+1}^{phy}$. Then, we can further calculate the attention feature maps *A*^*data*^ and *A*^*phy*^ based on these units:


(8) $$\begin{array}{l} \begin{array}{c} A^{data}=\text{softmax}\left(\frac{Q_{data}K_{data}^{T}}{\sqrt{d_{K_{data}}}}\right)\cdot V_{data}+{\text{X}}_{t+1}^{data},\\ A^{phy}=\text{softmax}\left(\frac{Q_{phy}K_{phy}^{T}}{\sqrt{d_{K_{phy}}}}\right)\cdot V_{phy}+{\text{X}}_{t+1}^{phy}, \end{array} \end{array}$$

where *d*_*K*_*data*__ and *d*_*K*_*phy*__ denote the dimensions of *K*_*data*_ and *K*_*pyh*_, respectively, and somfmax is an activation function. By doing so, we intend to learn the important information in the outputs from DDC and PGC separately, so as to guarantee the prediction performance.

Next, we attempt to capture the nonlinear relationships between ${\text{X}}_{t+1}^{data}$ and ${\text{X}}_{t+1}^{phy}$ by applying the cross-attention mechanism. The cross-attention feature map *CA*^*data*^ can be obtained by using *Q*_*phy*_ to query the key-value pair (*K*_*data*_, *V*_*data*_):


(9) $$\begin{array}{l} CA^{data}=\text{softmax}\left(\frac{Q_{phy}K_{data}^{T}}{\sqrt{d_{K_{data}}}}\right)\cdot V_{data}+{\text{X}}_{t+1}^{data}. \end{array}$$

Similarly, the cross-attention feature map *CA*^*phy*^ can be calculated as follows:


(10) $$\begin{array}{l} CA^{phy}=\text{softmax}\left(\frac{Q_{data}K_{phy}^{T}}{\sqrt{d_{K_{phy}}}}\right)\cdot V_{phy}+{\text{X}}_{t+1}^{phy}. \end{array}$$

Finally, all the feature maps obtained above are concatenated and fed into the output layer, to make the final prediction ${\tilde{\text{X}}}_{t+1}$:


(11) $$\begin{array}{l} {\tilde{\text{X}}}_{t+1}=F\left(concatenate\left(A^{data},A^{phy},CA^{data},CA^{phy}\right)\right), \end{array}$$

where *F* denotes the output layer in the Attention-based NLC. Same as MLP-based NLC, we also calculate the loss $loss_{NLC}=|{\tilde{\text{X}}}_{t+1}-{\text{X}}_{t+1}{|}^{2}$, to constraint the learning process.

#### 2.2.4 Objective function

The final optimization objective function, which takes account of both data and physics, is given as follows:


(12) $$\begin{array}{l} \begin{array}{c} min\left(w_{1}loss_{NLC}+w_{2}loss_{data}+w_{3}loss_{phy}\right), \end{array} \end{array}$$

where *w*_1_, *w*_2_, and *w*_3_ are hyper parameters.

## 3 Experimental results

In this section, we use six dynamical systems with different chaotic behaviors, i.e., the Rossler, Aizawa, Lorenz, Chua, Chen, and Halvorsen systems, which are widely used in chaotic systems dynamics prediction (Nasiri and Ebadzadeh, 2022; Cheng et al., 2021; Na et al., 2021; Wu et al., 2024; Kennedy et al., 2024; Gilpin, 2021), to validate the performance of the proposed PGL method in long-term forecasting of chaotic dynamics. We also perform an ablation study to analyze the contributions of different components of the proposed method to the chaotic dynamics prediction.

### 3.1 Descriptions of chaotic systems

#### 3.1.1 Rossler system

In 1976, Rössler (1976) proposed the well-known Rossler system, which exhibits chaotic phenomena and nonlinear dynamical behavior. The system is defined by the following differential equations:


(13) $$\begin{array}{l} \frac{dx}{dt}=-y-z,\\ \frac{dy}{dt}=x-ay,\\ \frac{dz}{dt}=b+xz-cz. \end{array}$$

#### 3.1.2 Aizawa system

In 1982, Aizawa and Uezu (1982) introduced a new chaotic system, which has multiple three-order nonlinear terms. The Aizawa system can be described by the following equations:


(14) $$\begin{array}{l} \frac{dx}{dt}=\left(z-b\right)x-dy,\\ \frac{dy}{dt}=dx+\left(z-b\right)y,\\ \frac{dz}{dt}=c+az-\frac{z^{3}}{3}-\left(x^{2}+y^{2}\right)\left(1+ez\right)+fzx^{3}. \end{array}$$

#### 3.1.3 Lorenz system

In 1963, Lorenz (1963) discovered the existence of a peculiar “butterfly effect” in meteorological systems when studying convective instability. The Lorenz system can be described by the following equations:


(15) $$\begin{array}{l} \frac{dx}{dt}=a\left(y-x\right),\\ \frac{dy}{dt}=cx-y-xz,\\ \frac{dz}{dt}=xy-bz. \end{array}$$

#### 3.1.4 Chua system

In 1986, Chua et al. (1986) introduced the Chua system, marking an advancement in the study of chaotic systems by linking chaos and nonlinear circuits. The equations of the Chua system are given as follows:


(16) $$\begin{array}{l} \frac{dx}{dt}=a\left(y-x-G\left(x\right)\right),\\ \frac{dy}{dt}=x-y+z,\\ \frac{dz}{dt}=-by,\\ G\left(x\right)=cx+\left(d+c\right)\left(\left|x+1|-|x-1\right|\right). \end{array}$$

#### 3.1.5 Chen system

In 1999, Chen and Ueta (1999) identified a chaotic attractor that bears similarities to the Lorenz system but is topologically distinct in their research on chaotic control. The Chen system can be described by the following equations:


(17) $$\begin{array}{l} \frac{dx}{dt}=a\left(y-x\right),\\ \frac{dy}{dt}=\left(c-a\right)x-xz+cy,\\ \frac{dz}{dt}=xy-bz. \end{array}$$

#### 3.1.6 Halvorsen System

The Sprott (2010) system, proposed by Arne Dehli Halvorsen, is a 3-D system of chaotic flows whose governing equations are cyclically symmetric and can be described as follows:


(18) $$\begin{array}{l} \frac{dx}{dt}=-ax-4y-4z-y^{2},\\ \frac{dy}{dt}=-ay-4z-4x-z^{2},\\ \frac{dz}{dt}=-az-4x-4y-x^{2}. \end{array}$$

All the above six dynamical systems have nonlinear and chaotic behaviors, posing great challenges for long-term prediction. We use the fourth-order Runge-Kutta method with a step size of 0.01 to obtain the chaotic time series containing 10, 000 steps, which are divided into training, validation, and testing datasets in a ratio of 6:2:2. Specifically, we utilize the data from the initial 6, 000 time steps for training purposes. This is followed by the subsequent 2, 000 time steps, which are designated for the validation process. Finally, we employ the data from the concluding 2, 000 time steps to test the performance of our model. Table 1 provides the details of system parameters and initial values. For parameters λ_1_, λ_2_, and λ_3_ in Equation 4 of the proposed method, we determine their values through a grid search strategy. Specifically, the parameter values are empirically constrained within the range of [0.05, 0.35], with a search step size of 0.05.

**Table 1:** The system parameters and initial values of six chaotic systems used in our study.

**System**	**Parameters**	**Initial values**
Rossler	*a* = 0.2, *b* = 0.2, *c* = 5.7	(*x*_0_, *y*_0_, *z*_0_) = (1.0, 1.0, 1.0)
Aizawa	*a* = 0.95, *b* = 0.7, *c* = 0.6, *d* = 3.5, *e* = 0.25, *f* = 0.1	(*x*_0_, *y*_0_, *z*_0_) = (0.1, 0, 0.1)
Lorenz	*a* = 10.0, *b* = 8/3, *c* = 28.0	(*x*_0_, *y*_0_, *z*_0_) = (1.0, 1.0, 1.0)
Chua	*a* = 15.6, *b* = 25.28, *c* = −0.75, *d* = 0.47	(*x*_0_, *y*_0_, *z*_0_) = (0.1, 0.1, 0.1)
Chen	*a* = 35.0, *b* = 3.0, *c* = 28.0	(*x*_0_, *y*_0_, *z*_0_) = (0, 1.0, 0)
Halvorsen	*a* = 1.4	(*x*_0_, *y*_0_, *z*_0_) = (1.0, 0, 0)

### 3.2 Comparison models and evaluation metrics

We select five representative methods as the baselines for performance comparison in our experiments. They are the long short-term memory (LSTM) (Hochreiter, 1997), the echo state network (ESN) (Pathak et al., 2017), the next generation reservoir computing method (NG-RC) (Gauthier et al., 2021), the knowledge-based neural ordinary differential equations method (K-NODE) (Jiahao et al., 2021) and DLinear (Zeng et al., 2023). Here, LSTM is a classic recurrent neural network model for time series prediction; ESN and NG-RC are representative methods specifically designed and widely used for chaotic system dynamics prediction; DLinear is a state-of-the-art deep learning method developed for complex time series forecasting; and K-NODE is a hybrid-learning approach which integrates the first principles knowledge, specifically the ordinary differential equations, with data-driven technologies, to predict chaotic systems dynamics. For LSTM, we use a three-layer architecture with a uniform hidden state size. To achieve its optimal performance, we experiment with a variety of hidden state sizes, specifically 8, 16, and 32, and report the best result. For ESN, we implement it with a spectral radius of 1.4 and a reservoir size of 300. For NG-RC and DLinear, we follow the default settings reported in their original papers. For K-NODE, we set the prior knowledge as the form of the governing equations with the approximated parameters learned by classic symbolic regression.

When assessing the effectiveness of the methods in capturing and forecasting the dynamical behavior of chaotic systems over the long term, it is a common practice to employ the model's own prediction as the input for forecasting subsequent time steps during the test phase. This iterative process can result in an increase in errors as the forecast horizon extends, especially in chaotic systems, where small deviations at the beginning can lead to significant differences in later outcomes. The mean absolute error (MAE), the root mean square error (RMSE), and the R^2^ (Amaranto and Mazzoleni, 2023) are used as evaluation metrics to measure the prediction performance. The MAE and RMSE are defined as follows:


(19) $$\begin{array}{l} \text{MAE}=\frac{1}{T}\overset{T}{\underset{t=1}{\sum }}|{ŷ}_{t}-y_{t}|, \end{array}$$

(20) $$\begin{array}{l} \text{RMSE}=\sqrt{\frac{1}{T}\overset{T}{\underset{t=1}{\sum }}{\left({ŷ}_{t}-y_{t}\right)}^{2}}, \end{array}$$

(21) $$\begin{array}{l} {\text{R}}^{2}=1-\frac{\overset{T}{\underset{t=1}{\sum }}{\left({ŷ}_{t}-y_{t}\right)}^{2}}{\overset{T}{\underset{t=1}{\sum }}{\left(ȳ-y_{t}\right)}^{2}} \end{array}$$

where ŷ_*t*_ denotes the predicted value of the model, *y*_*t*_ denotes the ground truth, ȳ represents the average value of the ground truth, and *T* is the corresponding forecast horizon.

### 3.3 Analysis of results

**Figure 2:**
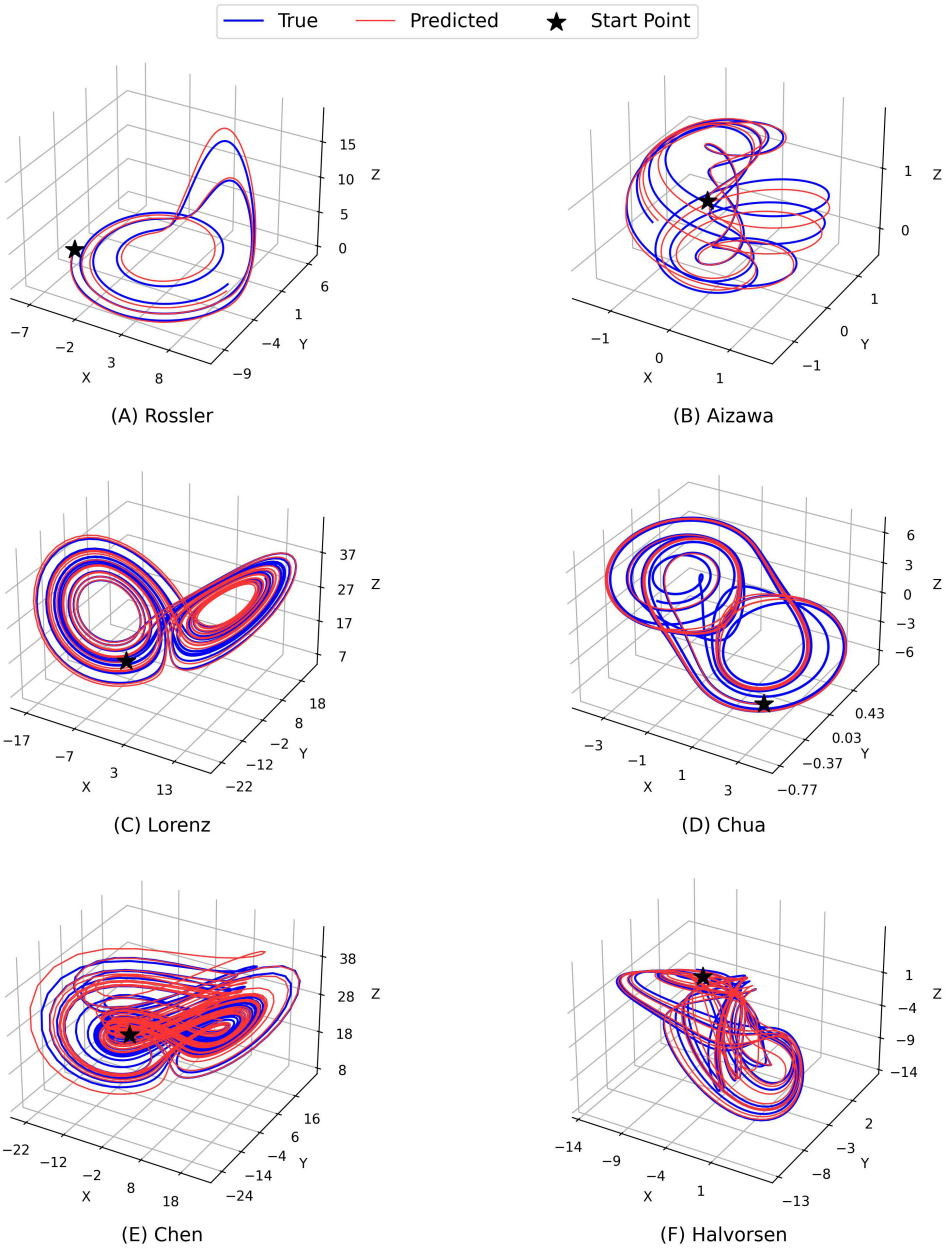
Comparison between the ground truth of dynamics of **(A)** Rossler, **(B)** Aizawa, **(C)** Lorenz, **(D)** Chua, **(E)** Chen, and **(F)** Halvorsen systems (blue) and the predictions generated by the proposed PGL-MLP method (red).

**Figure 3:**
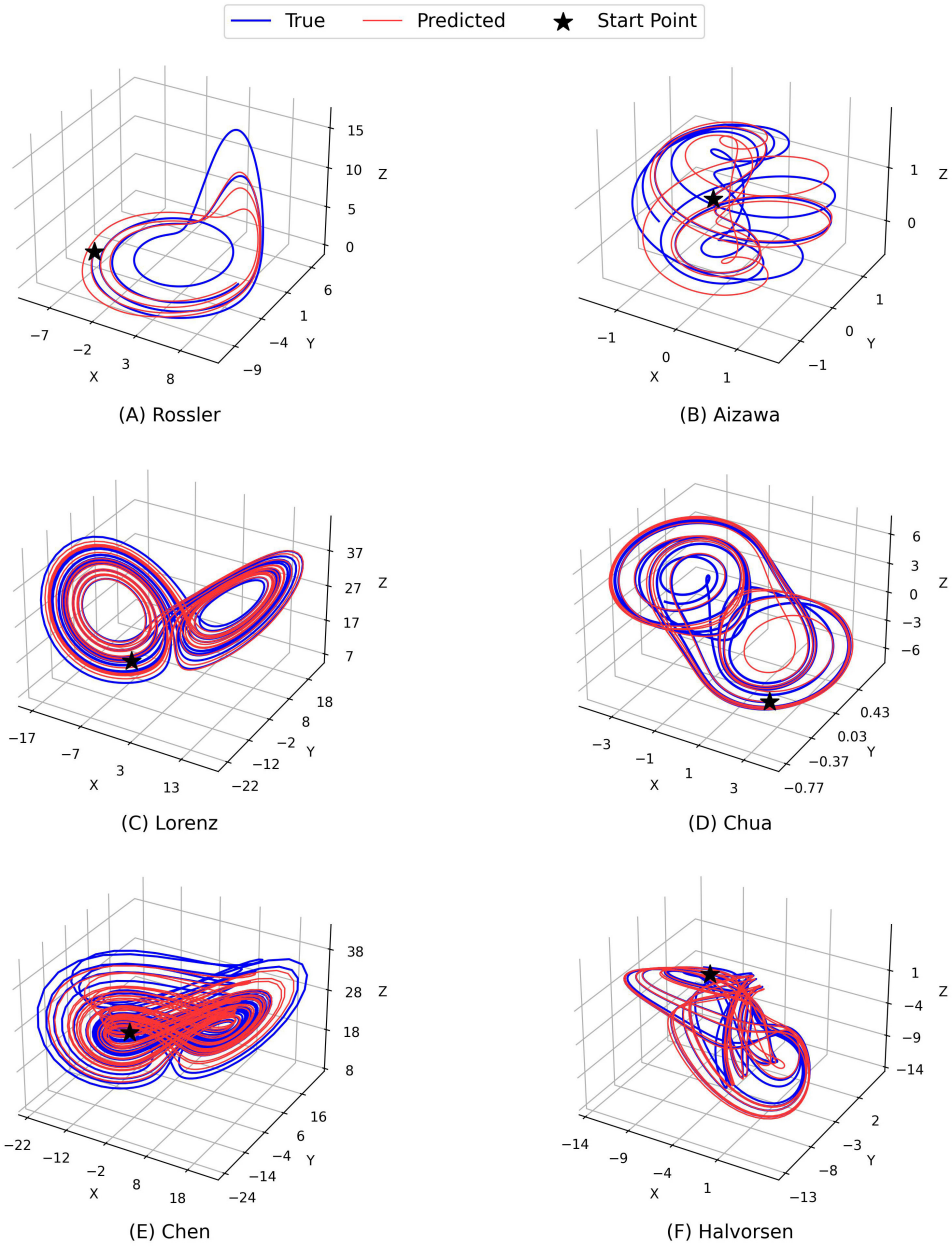
Comparison between the ground truth of dynamics of the **(A)** Rossler, **(B)** Aizawa, **(C)** Lorenz, **(D)** Chua, **(E)** Chen, and **(F)** Halvorsen systems (blue) and the predictions generated by the proposed PGL-ATT method (red).

Figures 2 and 3 demonstrate the comparison between the ground truth of dynamics of the Rossler, Aizawa, Lorenz, Chua, Chen, and Halvorsen systems in 2, 000 time steps, which is illustrated in blue in each sub-figure, and the predictions generated by the proposed PGL-MLP (Figure 2) and PGL-ATT (Figure 3) methods, which are shown in red. From these two figures, we can observe that both PGL-MLP and PGL-ATT can capture the dynamical patterns of these six chaotic systems. Although employing an iterative prediction process in the prediction phase brings great challenges to the task of long-term forecasting, the integration of data and physics enables our method to produce predictions that are consistent with actual dynamics.

**Figure 4:**
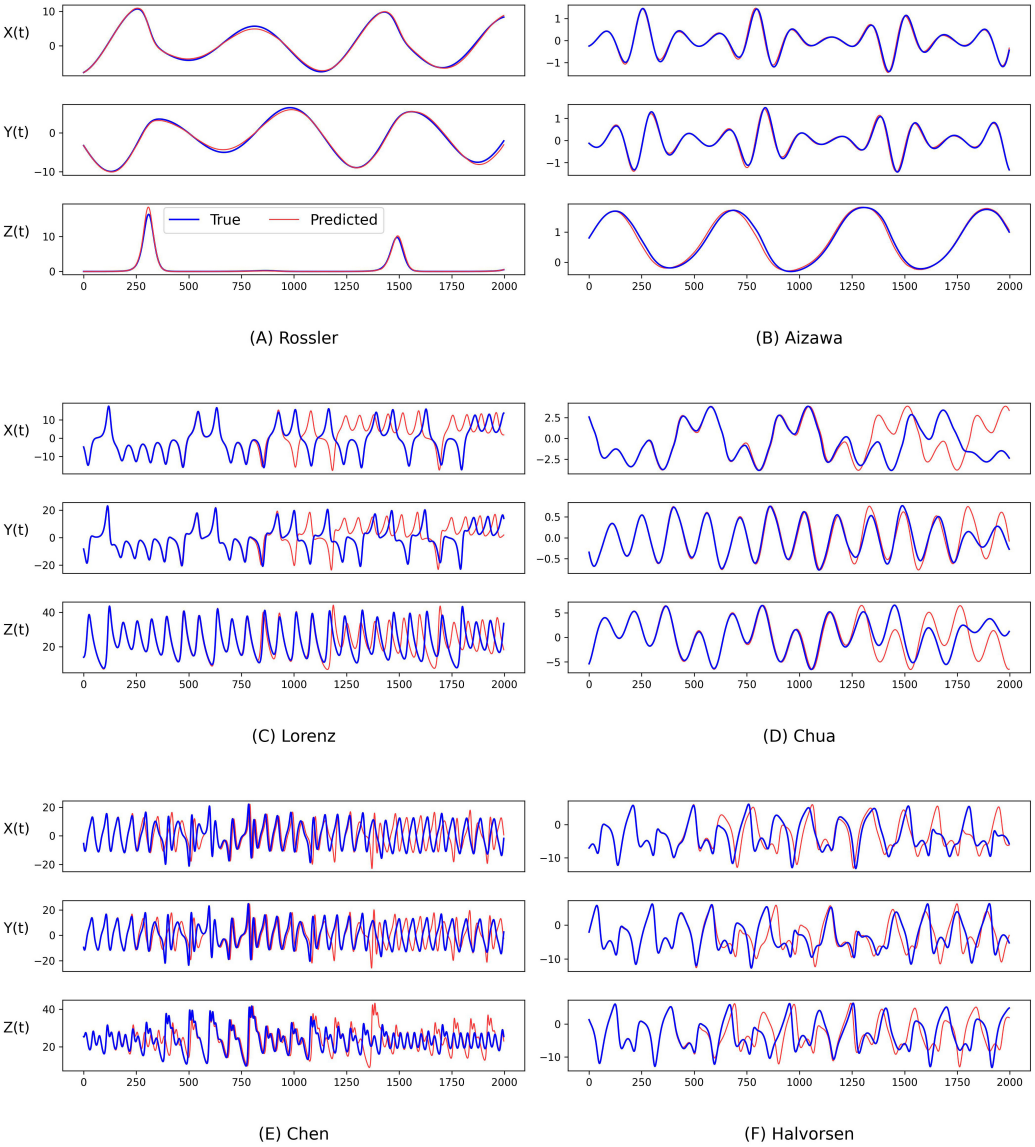
Comparison between the ground truth of the state variables of the **(A)** Rossler, **(B)** Aizawa, **(C)** Lorenz, **(D)** Chua, **(E)** Chen, and **(F)** Halvorsen systems (blue) and the predictions generated by the proposed PGL-MLP method (red) over time.

**Figure 5:**
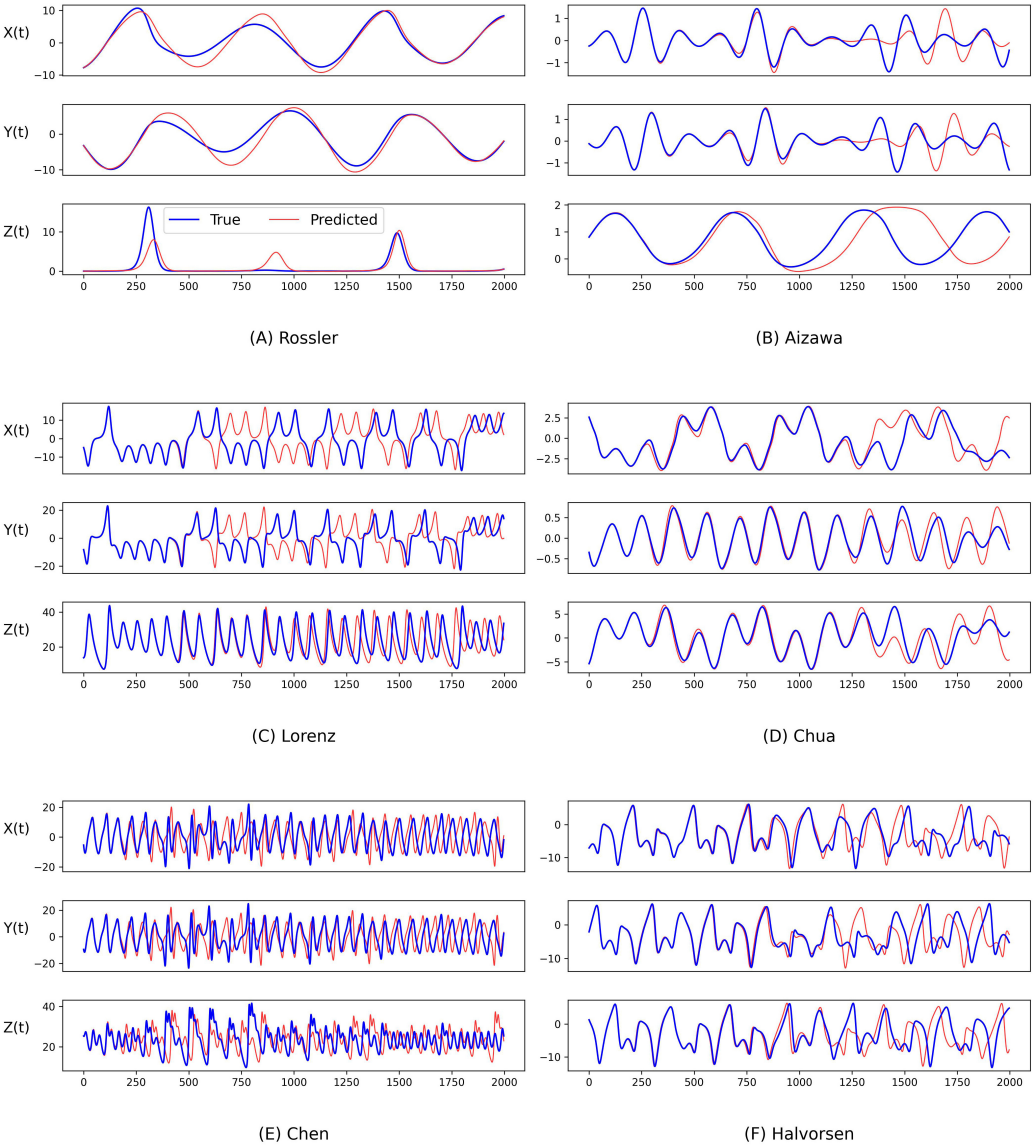
Comparison between the ground truth of the state variables of the **(A)** Rossler, **(B)** Aizawa, **(C)** Lorenz, **(D)** Chua, **(E)** Chen, and **(F)** Halvorsen systems (blue) and the predictions generated by the proposed PGL-ATT method (red) over time.

To further evaluate the performance of our predictions, we also conduct an analysis by visualizing the temporal evolution of the ground truth and predictions of the state variables in these chaotic systems in Figures 4 and 5. Generally, both PGL-MLP and PGL-ATT can make satisfactory predictions of the state variables *X*(*t*), *Y*(*t*), and *Z*(*t*) for these chaotic systems. However, the performance of each method on different systems varies slightly. For the Rossler system, the predicted curves of both PGL-MLP and PGL-ATT closely match the ground truth, accurately characterizing even the irregular patterns in *Z*(*t*) component; only one peak was missed by the PGL-ATT. This indicates that the proposed method successfully captures the dynamics of this chaotic system and thus is able to make accurate predictions in such a long-term period. For the Aizawa system, the PGL-MLP shows very good performance; its prediction is consistent with the ground truth in all 2, 000 steps. The performance of the PGL-ATT is also acceptable; the predicted dynamics match well with the actual curve in the first 1, 000 steps. For the Lorenz system, both PGL-MLP and PGL-ATT achieve high accuracy up to around 1, 100 time steps on the component *Z*(*t*), and 600 time steps on the components *X*(*t*) and *Y*(*t*), respectively. For the Chua system, PGL-MLP and PGL-ATT have similar performance, making accurate predictions up to about 1250 time steps, and then exhibit notable discrepancies in the components *X*(*t*), *Y*(*t*), and *Z*(*t*). Such discrepancies in Chen and Halvorsen systems appear earlier, compared with the Chua system. Interestingly, PGL-MLP's predictions for both the Chen and Halvorsen systems initially achieve high accuracy but subsequently exhibit noticeable disturbances. Fortunately, due to the model's ability to balance data and physical knowledge, it regains accuracy in its predictions after these disturbances.

To quantitatively compare the performance of our methods (i.e., PGL-MLP and PGL-ATT) with that of existing methods, we report the MAE and RMSE of all methods for different prediction horizons in Tables 2, 3, respectively. The results demonstrate that the proposed methods achieve the lowest prediction errors in most of the settings, demonstrating the effectiveness of our methods in making long-term predictions of chaotic system dynamics. An interesting observation is that the performance of PGL-MLP is generally better than that of PGL-ATT, despite the latter employing a more sophisticated attention mechanism. One potential explanation is that the complexity of the attention mechanism may lead to overfitting in the predictive model when compared to PGL-MLP. It is important to note that the task of predicting chaotic system dynamics differs from natural language processing, where attention mechanisms have demonstrated notable effectiveness. The former focuses on capturing the intrinsic, evolving patterns of dynamical systems, which may change over time, whereas the latter is primarily concerned with understanding consistent contextual relationships in input data. Consequently, a model that is overly complex or overfitted to historical data may not yield the expected performance in predicting chaotic systems dynamics.

**Table 2:** MAE of LSTM, ESN, NG-RC, DLinear, K-NODE, and the proposed PGL in different prediction horizons on six chaotic systems.

**Systems**	**Horizon**	**LSTM**	**ESN**	**NG-RC**	**DLinear**	**K-NODE**	**PGL-ATT**	**PGL-MLP**
Rossler	200-horizon	0.295	0.228	0.046	2.374	**0.039**	0.206	0.055
	600-horizon	0.934	1.411	0.865	4.318	0.322	1.264	**0.182**
	1,000-horizon	0.705	3.126	1.084	4.625	0.409	1.539	**0.236**
	1,400-horizon	0.583	3.919	1.199	5.111	0.481	1.341	**0.208**
	2,000-horizon	0.744	4.713	1.942	5.341	0.795	1.044	**0.225**
Aizawa	200-horizon	0.142	0.396	0.205	1.249	0.022	**0.011**	0.028
	600-horizon	0.198	0.649	0.419	1.104	0.034	**0.031**	0.049
	1,000-horizon	0.337	0.765	0.455	1.188	**0.058**	0.068	0.067
	1,400-horizon	0.444	0.807	0.497	1.292	0.103	0.137	**0.061**
	2,000-horizon	0.529	0.817	0.485	1.456	0.133	0.320	**0.060**
Lorenz	200-horizon	2.668	0.631	1.105	7.748	1.020	**0.245**	0.338
	600-horizon	4.828	5.462	5.088	6.602	3.618	**0.559**	0.616
	1,000-horizon	5.996	7.103	4.287	7.316	4.828	4.413	**1.189**
	1,400-horizon	6.579	8.110	5.285	7.541	5.964	5.915	**3.685**
	2,000-horizon	7.103	8.174	6.774	7.984	7.169	7.117	**5.536**
Chua	200-horizon	0.163	0.104	1.057	1.007	**0.008**	0.035	0.023
	600-horizon	1.372	0.999	1.815	1.321	0.095	0.253	**0.079**
	1,000-horizon	1.382	1.417	1.967	1.568	0.563	0.244	**0.117**
	1,400-horizon	1.242	1.855	2.047	1.622	1.042	0.321	**0.285**
	2,000-horizon	1.281	2.187	1.948	1.620	1.535	0.714	**0.912**
Chen	200-horizon	5.531	3.093	3.770	4.876	0.675	1.033	**0.300**
	600-horizon	8.085	6.480	7.906	6.182	6.017	5.936	**4.439**
	1,000-horizon	7.349	7.859	8.888	6.576	7.441	7.268	**4.526**
	1,400-horizon	8.026	8.323	8.731	6.604	7.682	7.152	**5.146**
	2,000-horizon	7.996	8.012	9.166	**6.484**	8.263	8.039	6.535
Halvorsen	200-horizon	0.456	1.168	0.434	2.167	0.063	**0.044**	0.107
	600-horizon	1.743	3.937	2.348	3.453	1.635	**0.217**	3.343
	1,000-horizon	1.515	5.155	2.872	3.575	1.241	1.991	**0.819**
	1,400-horizon	2.146	6.040	3.092	3.564	**1.297**	2.093	1.329
	2,000-horizon	3.207	6.642	3.655	3.610	**1.769**	2.486	2.550

The best performance in each setting is highlighted in bold.

**Table 3:** RMSE of LSTM, ESN, NG-RC, DLinear, K-NODE, and the proposed PGL-ATT and PGL-MLP in different prediction horizons on six chaotic systems.

**Systems**	**Horizon**	**LSTM**	**ESN**	**NG-RC**	**DLinear**	**K-NODE**	**PGL-ATT**	**PGL-MLP**
**Rossler**	**200-horizon**	**0.354**	**0.277**	**0.053**	**3.103**	**0.046**	**0.279**	**0.064**

	600-horizon	1.692	2.435	1.483	5.370	0.546	2.117	**0.315**
	1,000-horizon	1.371	5.050	1.637	5.562	0.610	2.295	**0.373**
	1,400-horizon	1.186	5.789	1.723	5.963	0.690	2.011	**0.331**
	2,000-horizon	1.352	6.670	3.209	6.133	1.318	1.175	**0.341**
Aizawa	200-horizon	0.187	0.508	0.242	1.597	0.029	**0.014**	0.043
	600-horizon	0.245	0.758	0.525	1.361	**0.044**	0.045	0.064
	1,000-horizon	0.451	0.907	0.563	1.416	**0.077**	0.099	0.088
	1,400-horizon	0.587	0.964	0.620	1.543	0.164	0.238	**0.081**
	2,000-horizon	0.673	0.975	0.600	1.741	0.202	0.513	**0.079**
Lorenz	200-horizon	4.511	0.922	1.624	9.884	1.733	**0.380**	0.561
	600-horizon	7.834	8.372	8.693	8.550	7.113	1.101	**0.891**
	1,000-horizon	8.900	9.667	7.773	9.410	8.131	8.266	**2.864**
	1,400-horizon	9.401	10.560	8.689	9.638	9.214	9.799	**7.405**
	2,000-horizon	9.967	10.595	10.067	10.050	10.15	10.632	**8.982**
Chua	200-horizon	0.209	0.123	1.398	1.274	**0.010**	0.040	0.027
	600-horizon	2.021	1.617	2.274	1.569	0.142	0.372	**0.111**
	1,000-horizon	1.992	2.038	2.402	1.854	1.162	0.339	**0.162**
	1,400-horizon	1.800	2.515	2.468	1.919	1.753	**0.503**	0.535
	2,000-horizon	1.795	2.855	2.350	1.918	2.264	**1.174**	1.495
Chen	200-horizon	8.174	5.487	5.591	6.229	1.071	1.536	**0.483**
	600-horizon	10.476	8.944	9.890	7.649	8.975	8.945	**7.298**
	1,000-horizon	9.738	10.251	10.824	8.165	10.134	9.915	**6.953**
	1,400-horizon	10.448	10.593	10.778	8.138	10.093	9.568	**7.680**
	2,000-horizon	10.151	10.322	11.147	**7.910**	10.641	10.335	9.124
Halvorsen	200-horizon	0.600	1.966	0.633	2.761	0.102	**0.064**	0.142
	600-horizon	2.686	5.912	3.323	4.342	2.822	**0.389**	0.529
	1,000-horizon	2.283	7.207	3.785	4.418	2.289	3.624	**1.427**
	1,400-horizon	3.142	7.999	3.953	4.414	2.234	3.449	**2.260**
	2,000-horizon	4.573	8.431	4.638	4.429	2.698	**3.666**	4.019

The best performance in each setting is highlighted in bold.

**Figure 6:**
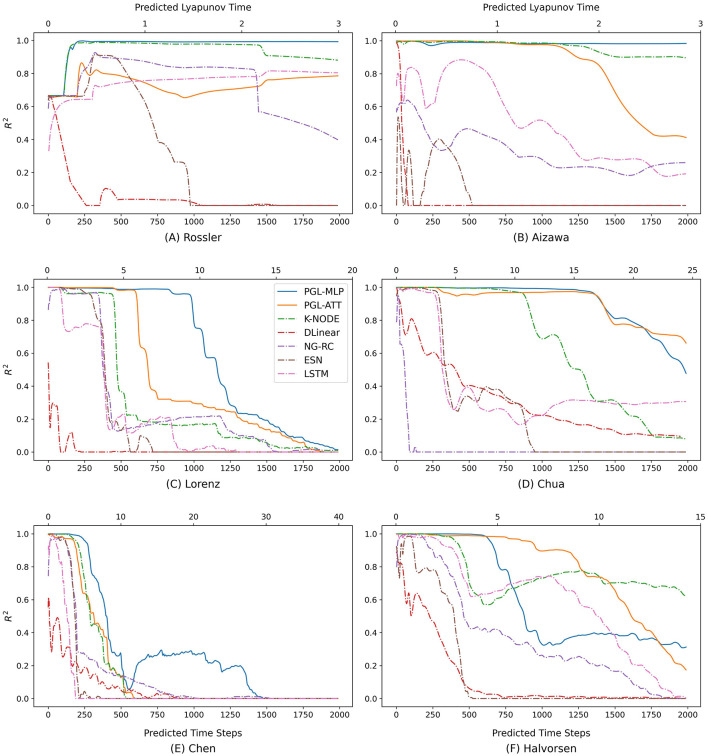
R^2^ of LSTM, ESN, NG-RC, DLinear, K-NODE, and the proposed PGL-ATT and PGL-MLP in different prediction horizons on six chaotic systems. **(A)** Rossler, **(B)** Aizawa, **(C)** Lorenz, **(D)** Chua, **(E)** Chen, and **(F)** Halvorsen.

In addition to the MAE and RMSE, we further analyze the performance of the comparison baselines and our proposed methods using the R^2^ metric, which ranges from 0 to 1 to indicate performance quality. As illustrated in Figure 6, we plot the R^2^ score's trend with increasing predicted time steps and calculate the specific Lyapunov Time for different forecasting horizons. The Lyapunov Time is a critical indicator of a system's chaotic behavior, representing the duration over which two initially close trajectories will diverge significantly (Sangiorgio and Dercole, 2020; Sangiorgio et al., 2021, 2022; Pathak et al., 2018; Patel et al., 2021; Vlachas et al., 2020). Our results show that the proposed methods achieve improved performance across the six chaotic systems. However, the performance of all methods varies across different chaotic systems. This variability is likely due to each system's unique Lyapunov Time, presenting different levels of prediction difficulty.

### 3.4 Ablation study

In this subsection, we conduct an ablation study to understand the individual contributions of the different components within our proposed method to predict chaotic dynamics. Specifically, we examine the performance of the Lorenz system dynamics prediction using four distinct configurations of our method: (1) employing only the DDC, which is an LSTM network; (2) integrating both DDC and PGC through a simple linear combination, referred to as PGL-Linear; (3) implementing the proposed method with attention-based NLC as described in this manuscript, referred to as PGL-ATT; and (4) implementing the proposed method with MLP-based NLC as described in this manuscript, referred to as PGL-MLP. In this ablation study, all experimental settings remain consistent with those used in previous experiments, including the initial conditions, the ratio of training and testing sets, and the prediction horizons.

Table 4 presents the results of the ablation study with respect to MAE and RMSE across various forecast horizons. The results obtained from the DDC alone exhibit relatively high MAE and RMSE across all prediction horizons. When integrating the DDC with the PGC using a simple linear combination (denoted as PGL-Linear), there is an observable improvement in performance compared to the DDC results. However, the enhancement achieved by PGL-Linear falls short of our expectations. One potential reason for this is that the relationship between the observational data and the physical principles governing the system's dynamics is likely nonlinear. As a result, a straightforward linear combination may be insufficient to capture the complexity of these interactions. This highlights the necessity of the proposed nonlinear combination (NLC) design for effectively integrating the DDC and PGC to enhance prediction accuracy. This necessity is further supported by the results from PGL-ATT and PGL-MLP, which demonstrate improved performance in terms of MAE and RMSE across all prediction horizons.

**Table 4:** MAE and RMSE of DDC, PGL-Linear, PGL-ATT, and PGL-MLP in different prediction horizons on the Lorenz system.

**Metrics**	**Horizon**	**DDC**	**PGL-Linear**	**PGL-ATT**	**PGL-MLP**
MAE	200-horizon	2.668	1.691	**0.245**	0.338
	600-horizon	4.828	3.389	**0.559**	0.616
	1,000-horizon	5.996	4.619	4.413	**1.189**
	1,400-horizon	6.579	5.506	5.915	**3.685**
	2,000-horizon	7.103	6.839	7.117	**5.536**
RMSE	200-horizon	4.511	2.823	**0.380**	0.561
	600-horizon	7.834	5.829	1.101	**0.891**
	1,000-horizon	8.900	7.540	8.266	**2.864**
	1,400-horizon	9.401	8.395	9.799	**7.405**
	2,000-horizon	9.967	9.493	10.632	**8.892**

The best performance in each setting is highlighted in bold.

## 4 Conclusion and discussion

In this paper, we proposed a physics-guided learning approach to predict the dynamics of chaotic systems. We experimentally evaluated the performance of our method on the Rossler, Aizawa, Lorenz, Chua, Chen, and Halvorsen dynamical systems. The experimental results demonstrated that our method outperforms other baselines in terms of prediction accuracy.

To our knowledge, PINN is among several representative techniques that employ neural networks to solve ordinary and partial differential equations. Other noteworthy methods include those based on the Deep Galerkin Method (DGM) (Sirignano and Spiliopoulos, 2018; Aristotelous et al., 2023) and Neurodifferential approaches (Lagaris et al., 1998; Ramuhalli et al., 2005), each offering unique contributions to the field. In our work, we utilize PINN as a typical example to demonstrate the efficacy of integrating data-driven structures with physical knowledge to accurately predict the dynamics of chaotic systems. This exemplification paves the way for further exploration into the integration of other physics-guided modules with data-driven components, potentially leading to enhanced predictive capabilities.

In our future work, we aim to extend our framework to scenarios where observations are noisy and the underlying governing differential equations are not fully known in advance. Moreover, in our current study, we used only six representative chaotic systems that exhibit distinct dynamical patterns such as the spiral-type chaos in the Rossler system (Rössler, 1977), the butterfly-shaped pattern in the Lorenz system (Li and Yin, 2009), and the double-scroll attractor in the Chua system (Chua, 2007) to demonstrate the feasibility of the proposed idea. Moving forward, we plan to conduct more comprehensive tests on 131 diverse chaotic systems across various domains (Gilpin, 2021)to further validate the robustness of our learning framework. Further, we intend to apply the proposed method to various real-world applications, such as infectious disease risk prediction, climate forecast, and traffic flow prediction. Additionally, we plan to conduct a comprehensive theoretical analysis of the proposed learning framework, attempting to quantitatively characterize its learning capacity and prediction error bounds using a series of key properties of chaotic systems, such as the Lyapunov Exponent and the Hurst Exponent.

## Data Availability

The raw data supporting the conclusions of this article will be made available by the authors, without undue reservation.

## Footnotes

^1^A preliminary version of this work appeared in the 4th French Regional Conference on Complex Systems (FRCCS 2024) (Feng et al., 2024).
