# Supplementary material for: Toward a physics-guided machine learning approach for predicting chaotic systems dynamics
Source: Front Big Data. 2025 Jan 17;7:1506443. doi: 10.3389/fdata.2024.1506443 (PMC11782262; doi:10.3389/fdata.2024.1506443)
Supplement: Supplementary file 1 [file Data_Sheet_1.pdf]

SUPPLEMENTARY MATERIAL

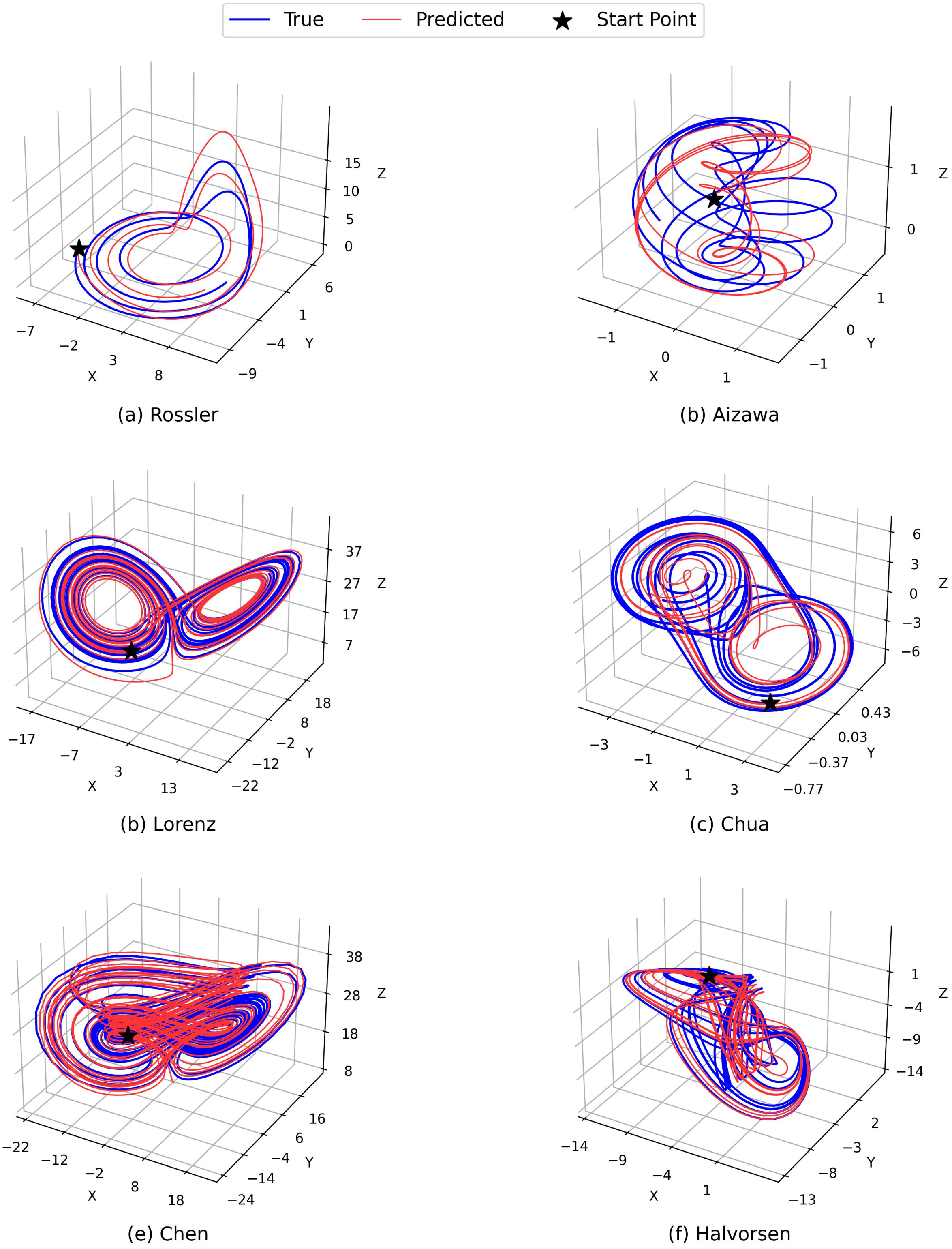

Figure S1. Comparison between the ground truth of dynamics of the Rossler, Aizawa, Lorenz, Chua, Chen, and Halvorsen systems (blue) and the predictions generated by the LSTM method (red).

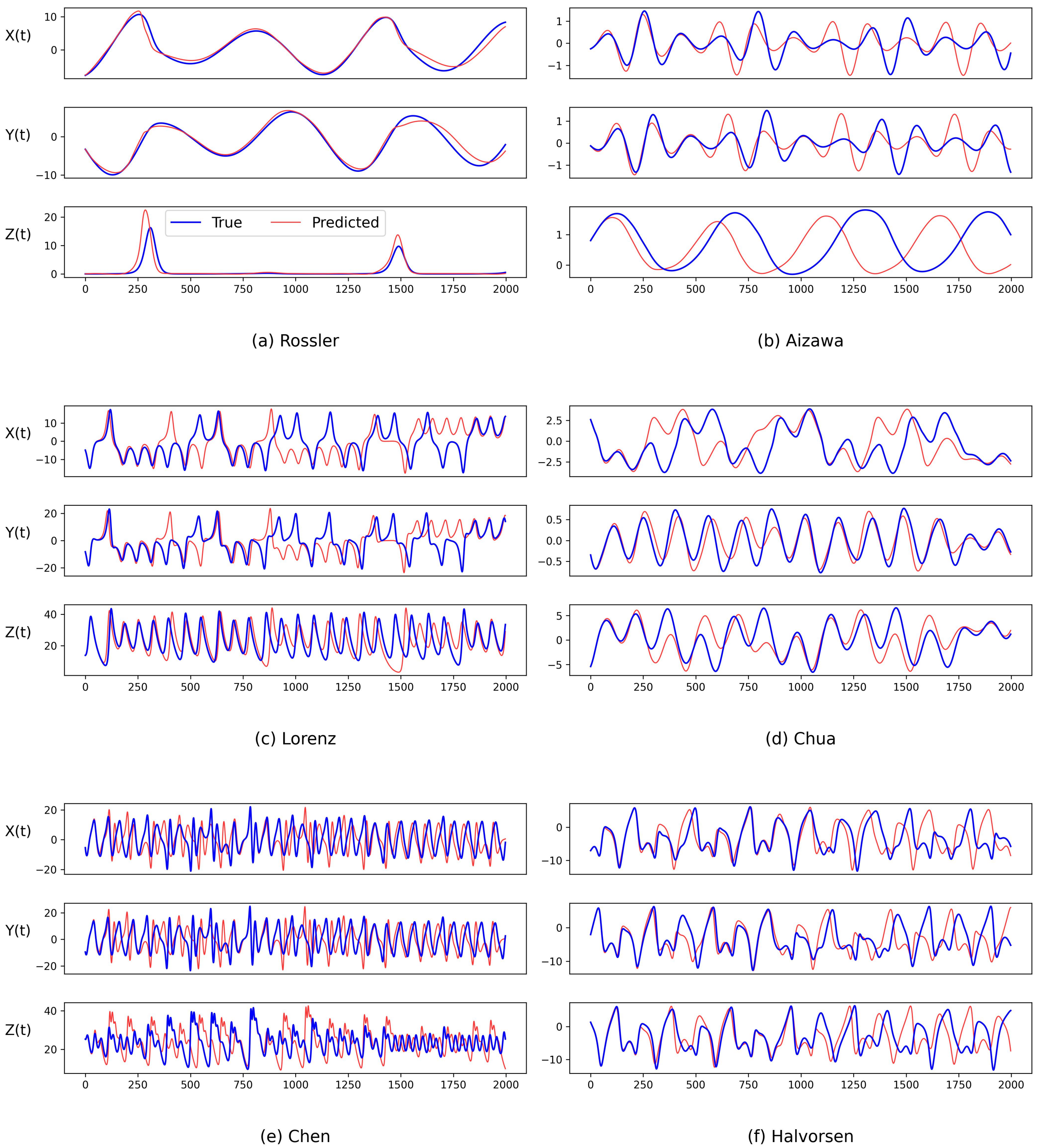

Figure S2. Comparison between the ground truth of the state variables of the Rossler, Aizawa, Lorenz, Chua, Chen, and Halvorsen systems (blue) and the predictions generated by the LSTM method (red) over time.

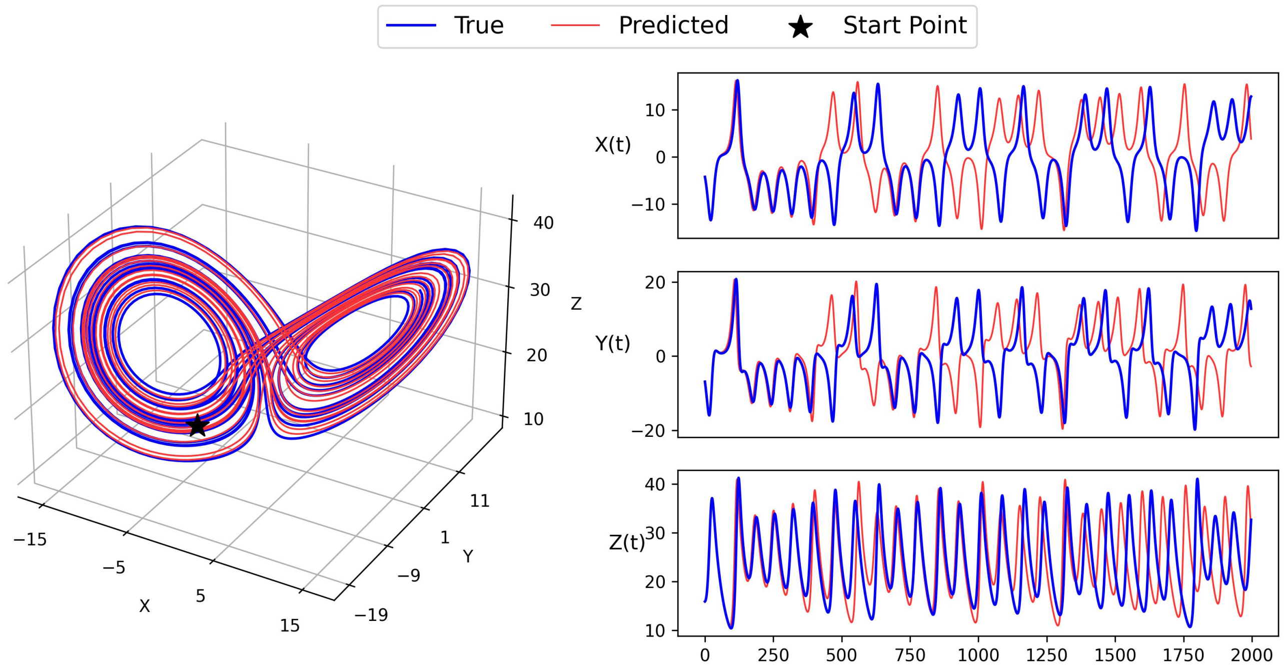

Figure S3. Comparison between the ground truth of the state variables of the Lorenz systems (blue) and the predictions generated by the PGL-Linear method (red).
